# Editorial: Advanced imaging in plants: exploring development and function

**DOI:** 10.3389/fpls.2026.1784311

**Published:** 2026-01-27

**Authors:** Agnieszka Ostrowska, Ján Kováč, Jaroslav Ďurkovič, Linnea Hesse

**Affiliations:** 1 The Franciszek Górski Institute of Plant Physiology, Polish Academy of Sciences, Kraków, Poland; 2 Department of Phytology, Faculty of Forestry, Technical University in Zvolen, Zvolen, Slovakia; 3 Institute of Wood Sciences, Department of Biology, Faculty of Mathematics, Informatics and Natural Sciences, University of Hamburg, Hamburg,, Germany

**Keywords:** advanced plant imaging, live imaging, multiscale analysis, non-destructive imaging, plant development, structure-function relationship

Plant development and function are highly complex, dynamic and multidimensional processes. Their analysis requires methodologies that combine stable and ideally natural, extrinsic and intrinsic conditions with repetitive or continuous non-invasive and non-destructive multiscale imaging. While advances in accessible computing have enhanced the applicability of complex imaging modalities in the biological sciences, further methodological development and optimization are required that are specifically tailored to plant research across spatial and temporal scales. Consequently, studies are emerging across all disciplines of plant science that focus on methodological innovation or improvements in image acquisition and processing often revealing complex and interdisciplinary approaches that enable groundbreaking insights into plant development and function.

Eight contributions in the Research Topic *Advanced Imaging in Plants: Exploring Development and Function* showcase how advanced instrumentation, experimentation, and computational analysis are revolutionizing our ability to study plants across scales.

Plant development has long been a central focus of plant biology research. Since the introduction of microscopy, studies have primarily documented plant structure and organ development through hand-drawn sketches. The advent of camera opened new possibilities for capturing developmental processes. Most of these results were obtained from a series of sections of organs at different developmental stages and the images were later reconstructed which was invasive and laborious leading to the inevitable destruction of the sample.

Modern techniques and protocols now allow continuous and non-destructive observation of developing plants, organs and tissues over the hours to years offering a more holistic understanding of developmental dynamics. However, maintaining living plants, organs and tissues under extended microscopical observation presents challenges due to limited space and relatively harsh conditions within microscopic setups.

A major challenge in methodological advancement in plant research still lies in achieving high-resolution imaging while maintaining natural physiological processes and simultaneously resolving spatial and temporal dynamics. As a result, diverse imaging techniques have emerged each tailored to specific research questions rather than offering a single, physically unattainable “holy grail” solution.

Singh Yadav and Roeder present an optimized live imaging and multiple cell layer growth analysis approach using *Arabidopsis* sepals, combining strong fluorescent membrane markers with reduced autofluorescence and semi-manual image processing. This methodology may serve as a comprehensive guide for live imaging of sepal development, and eventually other plant tissues, using confocal laser scanning microscopy.

Lee et al. demonstrate how a standard confocal microscope can be adapted for long-term, light-sheet–like time-lapse imaging using commercially available components, enabling automated tracking of growing roots and prolonged observation following laser ablation. Successful tracking of developmental events during root regeneration offers hope that a similarly simple and inexpensive procedure can be applied to study other developmental processes as well.

In wheat grain research, macroscopic analyses of whole organ sections could greatly assist in the identification of key grain regions and important developmental stages associated with cell wall chemical modifications. Multi-excitation hyperspectral autofluorescence imaging has previously been used to identify biological constituents in developing wheat grains. However, this method is constrained by a limited field of view and does not support the analysis of large sample sets.

Chateigner-Boutin et al. revealed that multispectral autofluorescence imaging combined with large principal component analysis provides a powerful way to study how tissue chemical composition and internal structural organization of plant organs vary at the macroscopic scale. This macroscopic approach allows whole-section analysis without fixation or labeling to reveal native autofluorescence signals, enabling robust statistical comparisons across samples.

Epicuticular wax accumulation and regulation of wax pathway gene expression during bioenergy sorghum stem development were investigated using scanning electron microscopy. Observations showed minimal wax deposition on the youngest internodes, while dense and diverse wax structures were found in mature internodes, including wax tubes, plate-like wax, and crystalline forms (Chemelewski et al.).

Wu et al. present correlative X-ray imaging as a novel, multimodal approach to investigate nanoparticle dissolution and nutrient transport in plants following foliar fertilization. By integrating small-angle X-ray scattering, X-ray fluorescence imaging, and micro-computed tomography, the authors were able to simultaneously characterize nanoparticle size and concentration, map elemental distributions, and embed these data within a three-dimensional representation of plant microstructure. This correlative strategy enabled the association of spatial and temporal attributes with nutrient translocation processes, revealing rapid nanoparticle dissolution after foliar application.

Rocha et al. applied cryoimmobilization techniques combined with transmission electron microscopy to achieve high-resolution ultrastructural analysis of microsporogenesis in *Rhynchospora*. The use of high-pressure freezing and freeze substitution significantly improved structural preservation, allowing the authors to uncover previously unresolved aspects of intracellular organization. Their results demonstrate that cytoplasmic architecture, endoplasmic reticulum organization, and cytoskeletal elements play a decisive role in nuclear selection during asymmetric microsporogenesis.

Naka et al. introduce an automated image analysis framework for petal segmentation in flower computed tomography datasets, based on a divide-and-conquer strategy and modern computer vision techniques. By segmenting petals in cropped two-dimensional regions and subsequently integrating the results into three-dimensional reconstructions, the authors overcame challenges associated with petal curvature and spatial overlap. This approach substantially reduces the need for manual segmentation and enables non-destructive, quantitative analysis of floral organ organization.

**Figure 1:**
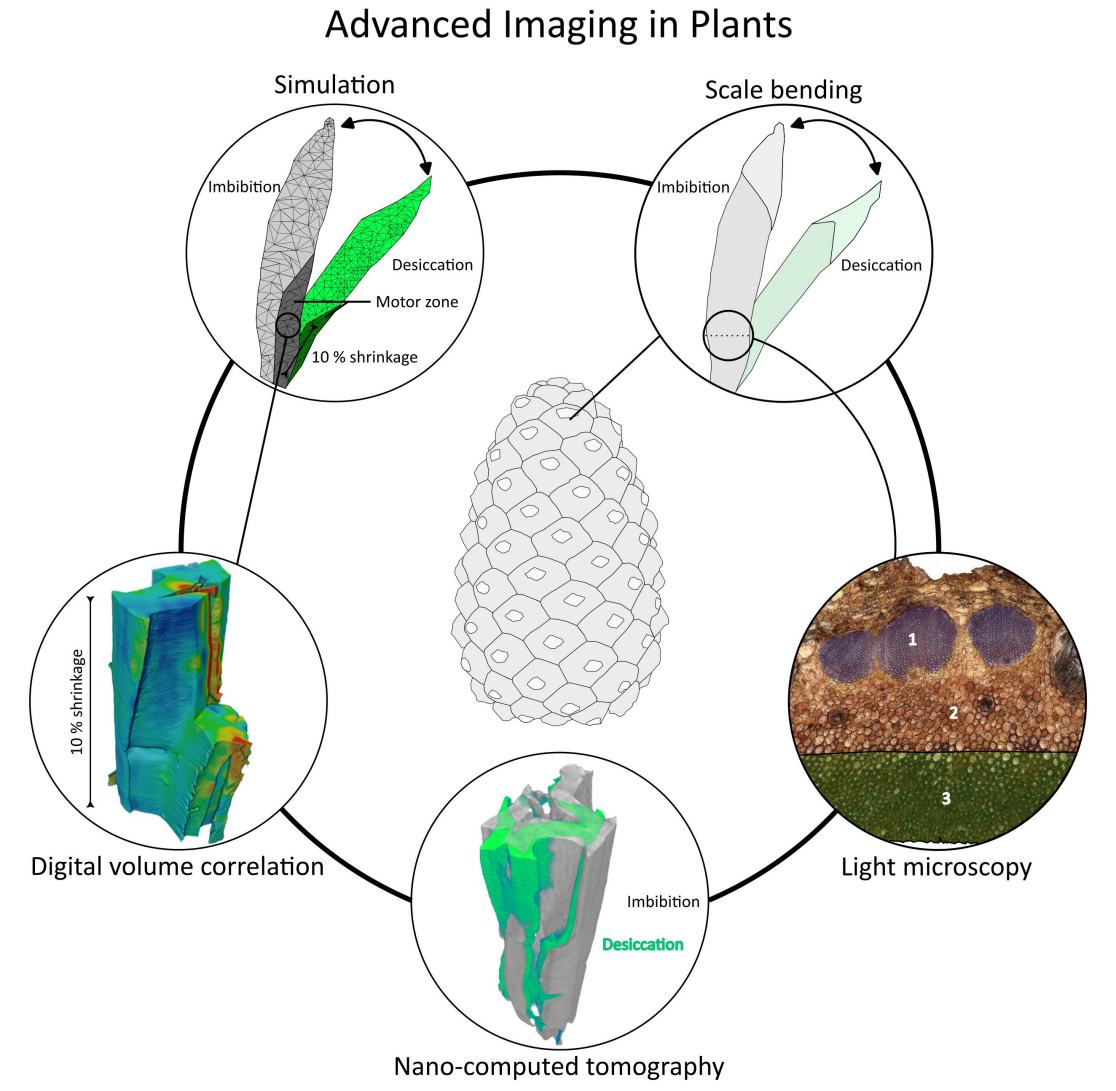
Pine cone scale bending is driven by a tri-layer tissue system, in which the sclereid cell layer within the motor zone of the scale (highlighted in green in the light micrograph) plays a dominant role. Desiccation-induced shrinkage of sclereid cells along their longitudinal axis (approximately 10%) causes the scale to bend downward. The contribution of cellular swelling and shrinkage to plant movement can be quantified using nano–computed tomography of cells in wet and dry states combined with digital volume correlation (DVC). DVC-based measurements of cell-wall volumetric changes provide quantitative parameters for finite element analysis of the underlying mechanical principle.

The multifunctionality of plants further complicates the clear identification of form–structure–material–function relationships for specific traits. This is particularly evident in lignocellulosic tissues of moving plant elements such as pine cone scales (**Figure 1**), explosive dehiscent fruits, or wood. Ulrich et al. highlight the methodological challenges in understanding hygroscopic cellular actuators involved in plant movements within biomimetic projects.

